# Characterization of the Mouse and Human Monoacylglycerol O-Acyltransferase 1 (*Mogat1*) Promoter in Human Kidney Proximal Tubule and Rat Liver Cells

**DOI:** 10.1371/journal.pone.0162504

**Published:** 2016-09-09

**Authors:** Shireesha Sankella, Abhimanyu Garg, Anil K. Agarwal

**Affiliations:** Division of Nutrition and Metabolic Diseases, Department of Internal Medicine and Center for Human Nutrition, UT Southwestern Medical Center, 5323 Harry Hines Blvd., Dallas, Texas 75390, United States of America; Jichi Medical University, JAPAN

## Abstract

Monoacylglycerol acyltransferase 1 (*Mogat1*) catalyzes the conversion of monoacylglycerols (MAG) to diacylglycerols (DAG), the precursor of several physiologically important lipids such as phosphatidylcholine, phosphatidylethanolamine and triacylglycerol (TAG). Expression of *Mogat1* is tissue restricted and it is highly expressed in the kidney, stomach and adipose tissue but minimally in the normal adult liver. To understand the transcriptional regulation of *Mogat1*, we characterized the mouse and human *Mogat1* promoters in human kidney proximal tubule-2 (HK-2) cells. *In-silico* analysis revealed several peroxisome proliferator response element (PPRE) binding sites in the promoters of both human and mouse *Mogat1*. These sites responded to all three peroxisome proliferator activated receptor (PPAR) isoforms such that their respective agonist or antagonist activated or inhibited the expression of *Mogat1*. PPRE site mutagenesis revealed that sites located at -592 and -2518 are very effective in decreasing luciferase reporter gene activity. Chromatin immunoprecipitation (ChIP) assay using PPARα antibody further confirmed the occupancy of these sites by PPARα. While these assays revealed the core promoter elements necessary for *Mogat1* expression, there are additional elements required to regulate its tissue specific expression. Chromosome conformation capture (3C) assay revealed additional *cis*-elements located ~10–15 kb upstream which interact with the core promoter. These chromosomal regions are responsive to both PPARα agonist and antagonist.

## Introduction

Triacylglycerol (TAG) is synthesized by two major pathways in mammals: the glycerol 3-phosphate (G-3-P) pathway [1] and the monoacylglycerol (MAG) pathway [2]. While the G-3-P pathway is ubiquitously present in tissues, the MAG pathway is tissue restricted to the small intestine, kidney, and adipose tissue but is minimally present in the adult liver [3]. Our interest in *Mogat1* increased while phenotyping the 1-acylglycerol phosphate O-acyltransferase 2 (*Agpat2*^*-/-*^) null mouse [4], a murine model of congenital generalized lipodystrophy type 1 (CGL1). The *Agpat2*^*-/-*^ mice revealed all the metabolic complications associated with human CGL1. The most interesting finding was a robust increase in the expression of *Mogat1* in the livers of *Agpat2*^*-/-*^ mice, both at the mRNA (~25–50 fold increase) and protein (~6-fold increase) levels [4]. The increase in the expression of hepatic *Mogat1* in *Agpat2*^*-/-*^ mice constitutes an alternate route by which the livers of these mice could synthesize TAG. Thus, *Mogat1* became a potential molecular target to reduce liver TAG synthesis. A few studies using siRNA or antisense oligonucleotides (ASO) in an acute setting showed suppression of *Mogat1* expression in mice livers [5–7], resulting in an increase in hepatic insulin signaling and sensitivity, improvement in whole body glucose homeostasis, and a modest decrease in liver TAG. However, using genetic approaches to delete *Mogat1* on the *Agpat2*^*-/-*^ and *ob/ob* genetic backgrounds did not result in any decrease in liver TAG levels [8]. However, these studies did not reveal how *Mogat1* is upregulated in the liver.

A limited number of studies related to the transcriptional regulation of *Mogat1* have been presented in the past [9, 10]. In these studies, ~2 kb promoter of human [9] and mouse [10] showed the significance of PPARγ in the transcriptional regulation of *Mogat1* in an *in-vitro* system. We initiated the current study in detail to help understand how the expression of *Mogat1* might be transcriptionally regulated in tissues like the stomach and kidney, in addition to the liver [3]. It is interesting to note that *Mogat1* is a highly conserved gene in mammals, it is highly expressed in tissues like the stomach and kidney [3] which are not known to synthesize any significant levels of neutral lipids, and that *Mogat1* might have a few non-enzymatic functions yet to be discovered. In this study, we characterized the promoter region of *Mogat1*, showing that the transcriptional regulation of *Mogat1* could be regulated by all three peroxisome proliferator-activated receptors (PPARs; α, γ and β/δ). The chromosome conformation capture (3C) assay further revealed that another putative PPAR regulatory region is located **~**10–15 kb further upstream of the transcriptional start site (TSS) which interacts with PPARα to regulate transcription of *Mogat1*.

## Experimental Procedures

Detailed experimental procedures used in this study are available upon request.

### Animals

Animals used in this study were approved by the Institutional Use and Care of Animals and BioSafety Committee (IACUC) at the UT Southwestern Medical Center.

### Bioinformatics for promoter analysis

Identification of transcription factor binding sites was performed using TESS-Transcription Element Search System (http://www.cbil.upenn.edu/tess/) and TRANSFAC (TRANScription FACtor database). “TRANSFAC^®^(www.biobase-international.com/transcription-factor-binding-sites) from BIOBASE Corporation” and [11]. Multiple sequence alignment was performed using the ClustalW2 program (http://www.ebi.ac.uk/Tools/clustalw2.index.html) and NCBI BLAST-basic local alignment search tool (http://blast.ncbi.nlm.nih.gov/Blast.cgi). Conserved regions between mouse, human and rat were identified using the University of California, Santa Cruz genome browser program (genome.ucsc.edu). To predict the presence of CpG islands in mouse and human Mogat1 promoters a bioinformatics online tool called Sequence Manipulation Suite was used [12]. For this analysis, ~500 bp of human or mouse *MOGAT1* upstream region and ~100 bp of downstream region was browsed.

### Cell lines

The cell lines included in this study were human embryonic kidney 293 (HEK-293), human kidney proximal tubule (HK-2), human epithelial colorectal adenocarcinoma (Caco-2), human colorectal adenocarcinoma (HT-29), human hepatocarcinoma (Huh-7), human breast adenocarcinoma (MCF-7), mouse fibroblasts (3T3-L1), Chinese hamster ovary (CHO), rat hepatic tumor (HTC) and normal rat kidney (NRK). Most of the cells used were obtained from ATCC and a few were obtained from other investigators on the campus.

### Cell culture

HEK-293, Caco-2, Huh-7, HTC and HT-29 were grown in high glucose DMEM supplemented with 10% FBS and 1% antibiotics. HK-2 cells were grown in low glucose DMEM. CHO cells were grown in F-12 media and HT-29 cells were grown in McCoy’s 5a media with 10% FBS maintained in a 37°C incubator with 5% CO_2_. All cell culture reagents were from Life Technologies (Grand Island, NY).

### Quantitative real-time PCR

Primers for mouse genes used in conventional and real time PCR reactions in this study are listed in Table 1. All RT-qPCR was carried out in 20 μl reaction volume in 96-well plates using the ABI PRISM 7700 sequence detection system as reported previously [4]. All human genes were amplified using Taqman master mix and primer/probe sets (MOGAT1-Hs00369695_m1, PPARα-Hs00231882_m1, PPARγ-Mm01184322_m1 and PPARδ-Hs00606407_m1, 18S-4333760F) (Life Technologies). Human and rat transcripts were normalized to 18S and mouse transcripts to cyclophilin.

**Table 1:** Primers used in this study.

RACE and amplification of full length transcript			
Primer name	Primer Sequence	Primer name	Primer Sequence
mMOGAT1 +194F	CAGAGCAAGGAGGCAGAAGA	mMOGAT1_5UTR_F	CAAATCCTGCGAAAGGAGTC
mMOGAT1 +457R	GGAACAACGGGAAACAGAAC	mMOGAT1_exon6_R	CTGAGGTCGGGTTCAGAGTC
mus_GAPDH_1F	GGAGCCAAAACGGGTCATCATCTC	mMOGAT1_5UTR_F_2	CCTCCCAGTCGGTAGCAGTA
mus_GAPDH_1R	GAGGGGCCATCCACAGTCTTCT	mMOGAT1_F_1	GGAGGTGGCAATGTCTCAATC
mMOGAT1_R_1	CATCTTCTGCCTCCTTGCTC	mMogat1 sybr 66 F	GCAGTGGGTCCTGTCCTTC
mMOGAT1_R_2	AGGAAGGACAGGACCCACTG	mMogat1 sybr 221 R	CAGTTCCATCTTCTGCCTCCTT
hMOGAT1 F	CATACCCCAGAGCGAGGAG	Cyclo sybr 334 F	TGGAGAGCACCAAGACAGACA
hMOGAT1 R	CGAAAGACAGGACACCAGAA	Cyclo sybr 335 R	TGCCGGAGTCGACAATGAT
hMOGAT1_CDS_F +523	AAGGAGGGAGGTGGAAACAT	hMOGAT1_Exon_R_1	AGCAGCAGGTATTTCAGGAC
hMOGAT1_CDS_R +786	AAACAGGGGCAAAGCAAAC	hGAPDH 361 F	ATCTCTGCCCCCTCTGCT
hMOGAT1_3’UTR_F	CCAGCGGAAAGGATTTGTTA	hGAPDH 793 R	CCTGCTTCACCACCTTCTTG
hMOGAT1_3’UTR_R	CAAAGCAAACCCCATGATCT	hMOGAT1_CDS_F_1	GATCATGGGGTTTGCTTTGC
hMOGAT1_Exon_R	GGCTCCAACTGCCATTATTC	hMOGAT1_Exon_F	GGCCATGAAGGTAGAGTTTG
rMOGAT1_5’UTR_F	TAGTTCCTTCCTTCTCCGCC	rMOGAT1_Exon4_F	AAGGAGGTGGGAACAT
rMOGAT1_5’UTR_2F	ACAGACCCGGAGAAAGGAGT	rMOGAT1_Exon4_R	ATGGGTCAAGGCGATCTTA
rMOGAT1_Exon1_F	ATGATGGTTGAGTTCGAGCCA	rMOGAT1_Exon_R	CACTTTGTCTTGGGCAGTCC
rMOGAT1_Exon1_2F	GGATCCTGGTCATCTTGGTG	rMOGAT1_Exon6_R	CATCAATCTGCTCTGGGGTC
rMOGAT1_Exon2_R	TCCAGTTCTGAACCCAGCTC	rMOGAT1_Exon6_1R	GTATGGCTTGGAGGAAGACG
rMOGAT1_Exon3_R	GAACAACGGGAGCCAGAAC		
**Promoter constructs and deletion mutagenesis**			
mMOGAT1_R	GGGACTGTGTCTGGTGAGTG	mMOGAT1_16kb_up_F	ATGCTCTTTTCCTATGCTTAACTCC
mMOGAT1_6kb_up_F	CAGGGATCAAATGTAGGTCTTGAG	mMOGAT1_4kb_up_R_1	GACCATGTGTGGATATAGACTGTGTG
mMOGAT1_4kb_up_R	CACAGAGGCCAGAAGAGGTATTG	mMOGAT1-5371_F	CACTGGTGGAATGTCTCTCC
mMOGAT1-3473_F	AGAGCTGGTTTTGCTTCCTG	mMOGAT1-5022_F	GGATAGTGTGAAAATTCGCTTG
mMOGAT1-3094_F	GAGTAACCAAAATGGCTGCAT	mMOGAT1-4601_F	CTGGATGGTTCCAAGGGTTA
mMOGAT1-2832_F	CATTGGTGGCTTCCTGTTAG	mMOGAT1-4185_F	TGCTTCTAGGTATGATGGAGGA
mMOGAT1-2340_F	TTCGTTCATGCCCAATATCC	mMOGAT1-3807_F	GCCAGGGCTACCTAACAAAA
mMOGAT1–4185_R	TCCTCCATCATACCTAGAAGCA	mMOGAT1_prom_F	GGCTGGAAAGACAGCTCAAC
mMOGAT1_prom_del_F	CCTGCCCCCCCCCCAAAATTTTCTCACAAGATAAT	mMOGAT1_prom_R	GGGCAGAGAAGTCATACAGG
mMOGAT1_prom_del_R	ATTATCTTGTGAGAAAATTTTGGGGGGGGGGCAGG		
**ChIP assay**			
mMOGAT1_ChIP_F	TTGTCCCACCGGATTCTAAC	hMOGAT1_ChIP_1.1F	GCTTGGGCTCCATATCCTC
mMOGAT1_ChIP_R	GCACGGGTCTCTCTTTTTGT	hMOGAT1_ChIP_1.1R	AGTCGCCAAAAGTCATCGAG
mMOGAT1_ChIP_F1	GCACATACGAGGCAGAGACA	hMOGAT1_ChIP_1.2F	CTGAGGACCCAAGAGTGAGG
mMOGAT1_ChIP_R1	GGATATTGGGCATGAACGAA	hMOGAT1_ChIP_1.2R	GGGAGAGGGGTCAACTGG
mMOGAT1_ChIP_F2	TGCAGACTTCTTAAACTGCTAAGG	hMOGAT1_ChIP_1.3F	GACTCGCCTCTCCAGGTTTT
mMOGAT1_ChIP_R2	TGGGCCTAATTGACTCCAA	hMOGAT1_ChIP_1.3R	ATCTTCCCCCAGTGCTAGGT
mMOGAT1_ChIP_F3	TAGGGTGGCAAATCCTGGTA	mMOGAT1_ChIP_R3	TGACTCTGGTGCAATATGCTG
**3C assay**			
hMOGAT1_3C_F1	ATGCTTCCAGGGCGCTAAC	hMOGAT1_3C_R3	TGGTGGGCCTGAAGAGAATA
hMOGAT1_3C_R1	GATTTGCACACCTTTCTCTGC	hMOGAT1_3C_F4	GGTCTTTCTTAATCCCAAGATTTTT
hMOGAT1_3C_F2	TCAGCAGAAATTAAGGAGATGC	hMOGAT1_3C_R4	GCTGGTTCATTCACTCACTCC
hMOGAT1_3C_R2	TGGGTAGTAGGATATGGACATGC	hMOGAT1_3C_F5	AGACAATCTGGCCTGTGACC
hMOGAT1_3C_F3	CCCTGAGAGCTGAAGAGGTG	hMOGAT1_3C_R5	TGAAAATGTACCAAGCACCA
**PPARs**			
rMOGAT1_PPARα_F	TCACACAATGCAATCCGTTT	rMOGAT1_PPARγ_R	ACTGGCACCCTTGAAAAATG
rMOGAT1_PPARα_R	GGCCTTGACCTTGTTCATGT	rMOGAT1_PPARβ/δ_F	AGGACATGAGCCATCCAAAG
rMOGAT1_PPARγ_F	CCCTGGCAAAGCATTTGTAT	rMOGAT1_PPARβ/δ _R	TACACCCCTTCCCTTCAGTG

### Northern blot analysis

Northern blot analysis was performed as previously described [13]. Twenty μg of total RNA was separated on a 1% agarose–formaldehyde denaturing gel and hybridized with ^32^P-labeled DNA probe (a 250 bp cDNA fragment of Mogat1). Membranes were washed with hybridization buffer at 50°C for 1 h, followed by 2xSSC/0.5% SDS at 50°C for 30 min. The blots were exposed to X-ray film.

### 5’ rapid amplification of cDNA ends (5’ RACE)

The 5′ region of the transcript was amplified using 5′-RNA ligase mediated RACE kit as suggested by the manufacturer (Ambion) to generate the 5′ cDNA sequences of the *Mogat1* gene. The first round of PCR was performed using 5′-Outer and 5′ Outer Universal Primers followed by the second round of PCR using 5′-Inner and 5′ Inner Universal Primers (Table 1).

### Reporter gene constructs

The mouse *Mogat1* upstream region was cloned into the pGL3-basic luciferase reporter vector and was used to generate serial deletion promoter constructs. The deletion clones were amplified and sequenced to determine PCR errors. Primers used are listed in table 1.

### Transient transfection in cells

HK-2 or HTC cells were seeded at a density of 3 x 10^5^ cells in a 6-well plate. The cells were transfected with a 1:3 ratio of plasmid DNA:fugene HD complex (2 μg pGL3 Basic or various promoter constructs, pGL3-Mogat1 plasmid, along with100 ng pRL Luc (renilla luciferase control vector). 24 hr post-transfection cells were washed with 1X PBS. Luciferase reporter assay was performed using dual luciferase reporter assay system according to the manufacturer’s protocol (Promega, Madison, WI). The ratio for firefly:renilla luciferase activity was calculated and the relative luciferase activity was normalized to protein and expressed as a fold change in relative luminiscence units (RLU).

### Deletion of PPAR sites in the Mogat1 promoter

The plasmid consisting of Mogat1 promoter region from -2832/+136 bp (nucleotides numbered from the translational codon ATG, where adeneine is +1) was used as the parental clone for all mutagenesis experiments. Mutation in PPRE site 1 and site 2 located at -592 and -2518 bp of the promoter were generated using Quikchange XL site directed mutagenesis kit (Agilent) according to the manufacturer's protocol. All the deletion constructs were verified by Sanger sequencing. Primers used are listed in Table 1.

### Chromatin immunoprecipitation (ChIP) assays in whole mouse kidney and in HK-2 cells

ChIP assays were performed as reported earlier [14]. Briefly, ~300 mg of frozen or fresh mouse kidney from 2–3 wk old female mice was homogenized and sonicated to shear DNA to an average fragment size of ~200–1000 bp. An aliquot of the sonicated sample was saved as an input. Protein A/G beads (Santa Cruz Biotechnology, Dallas, TX) were added to all samples and incubated for 1 hr at 4°C to pre-clear. To the immunoprecipitate DNA-protein complexes (IP), 2 μg of PPARα antibody (H-98, Catalog # sc-9000, Santa Cruz Biotechnology) was added. The IP DNA was eluted and the samples were then incubated with 5M NaCl for 4–5 hr at 65°C to reverse the cross-links. DNA was purified and PPARα binding PPRE was amplified using the primers shown in Table 1.

HK-2 cells were seeded at a density of 1 x 10^7^ cells per 150 mm plate. Cells were crosslinked with 37% formaldehyde to crosslink (1% final conc) and incubated at room temperature for 10 min, then 1.25 M Glycine (0.125 M final conc) was added to quench unreacted formaldehyde. Cells were scraped with ice-cold PBS containing protease inhibitors and pelleted. For pre-clearing, 100 μl of protein A/G agarose beads were added and incubated at 4°C for 1 hr with rotation. The beads were pelleted at 300xg for 5 min at 4°C. The supernatant was removed and protein A/G agarose beads along with 2 μg of PPARα antibody were added and incubated overnight at 4°C with rotation. Protein A agarose beads were pelleted. The immune complexes were eluted in elution buffer and digested with proteinase K. The DNA was recovered by phenol/chloroform extraction and ethanol precipitation. The purified DNA is used for PCR as above.

### Treatment of HK-2 and HTC cells with PPAR agonists and antagonists

HK-2 cells were seeded at a density of 1x10^6^ per well in a 6-well plate and the cells were transfected with Mogat1_-2832/+136 construct. 24 hr post transfection cells were treated with DMSO or 25, 50 or 100 μM WY14643, GW6471, rosiglitazone, GW9662, GW0742 or GSK0660. In some experiments, to measure the competitive inhibition of PPARs by their respective antagonist, cells were incubated with an equimolar concentration of agonist and antagonist for respective PPARs. HTC cells were also transfected as above and treated with 100 μM of the PPAR agonists and antagonists. After 24 hr of treatment, cells were lysed in passive lysis buffer (Promega) and luciferase activity was measured using dual luciferase reporter assay kit from Promega according to the manufacturer’s protocol. The ratio for firefly:renilla luciferase activity was calculated and the relative luciferase activity was normalized to protein and expressed as a fold change in RLU.

### Treatment of primary mouse hepatocytes with BSA conjugated fatty acids

Primary mouse hepatocytes were isolated from 1 month-old non-fasted WT and *Agpat2*^*-/-*^ female mice as described earlier [15]. 1x10^6^ cells were seeded into a collagen coated 6-well plate and allowed to attach for 2 hr in low glucose DMEM containing 10% FBS and 1% antibiotics. A stock solution (5 mM) of the following fatty acids were prepared by conjugating these fatty acids to BSA as described earlier: palmitic, stearic, oleic and arachidonic acids [16]. The solution was filter sterilized with a 0.2 μm syringe filter and added to the cells at the desired concentration. After 2 hr cells were lysed using RNA STAT-60. cDNA was made and RT-qPCR was performed to measure the expression of Mogat1.

### Chromosome Conformation Capture (3C) Assay in HK-2 cells

The 3C assay was performed according to the method described earlier [17]. Briefly, HK-2 cells were seeded in a 15-cm dish and grown to 90% confluency. Cells were washed with ice-cold PBS and crosslinked with 2% formaldehyde for 10 min at room temperature (RT). The cross-linking was terminated by adding 0.125 M glycine and cells were lysed for 1 hr with ice-cold lysis buffer containing 10 mm Tris (pH 8.0), 10 mm NaCl, 0.2% Nonidet P-40, and 1 mm dithiothreitol. The nuclei of the cells were then harvested and suspended in the appropriate restriction enzyme buffer containing 0.3% SDS and incubated at 37°C for 1 hr with gentle shaking. SDS was then sequestered from the samples by the addition of 1.8% Triton X-100 and incubated at 37°C for 1 hr. The samples were digested with BglII at 37°C for 16 hr. BglII was inactivated by the addition of 1.6% SDS and incubated at 65°C for 20 min. Samples were diluted with T4 DNA ligase buffer (Life technologies) to achieve ∼3 ng DNA/μl, and then incubated with 200 units of T4 DNA ligase for 4 hr at 16°C. Samples were incubated with Triton X-100, followed by incubation with Proteinase K (200 μg/ml) at 65°C for 16 hr to reverse the cross-linking. This was followed by the addition of 10 μg of RNase/ml, and the DNA was purified by phenol/chloroform extraction. 300 ng of DNA was then analyzed by 40 cycles of touchdown PCR with Failsafe polymerase (Epicenter, Madison, WI).

The PCR products were resolved on a 2% agarose gel and the amplified DNA was gel purified using the QIAquick gel extraction kit (Qiagen) and then cloned into pDrive cloning vector (Qiagen). The clones were sequenced to confirm the ligation of the two distal fragments. RT-qPCR was performed to quantify crosslinking efficiency. To determine the role of PPARα, cells were also incubated with DMSO, WY16873 (100 μM) or GW6471 (100 μM) for 24 hr prior to the 3C assay. Primers are listed in Table 1. Similarly, 3C assay was also performed in HTC cells. Based on the conveniently located *HindIII* restriction sites in the ~20 kb *Mogat1* promoter of rat, *HindIII* was chosen as an appropriate restriction enzyme for rat promoter 3C assay. Primers used for 3C assay in HTC cells are listed in S1 Table.

### Statistical analysis

Values are given as mean ± S.E.M. Statistical significance was calculated by two-tailed Student’s t-test using GraphPad Prism version 6.04 for Windows. A p-value ≤0.05 was considered statistically significant.

## Results

### Selection of an appropriate cell culture model system to study *Mogat1* promoter

**Fig 1:**
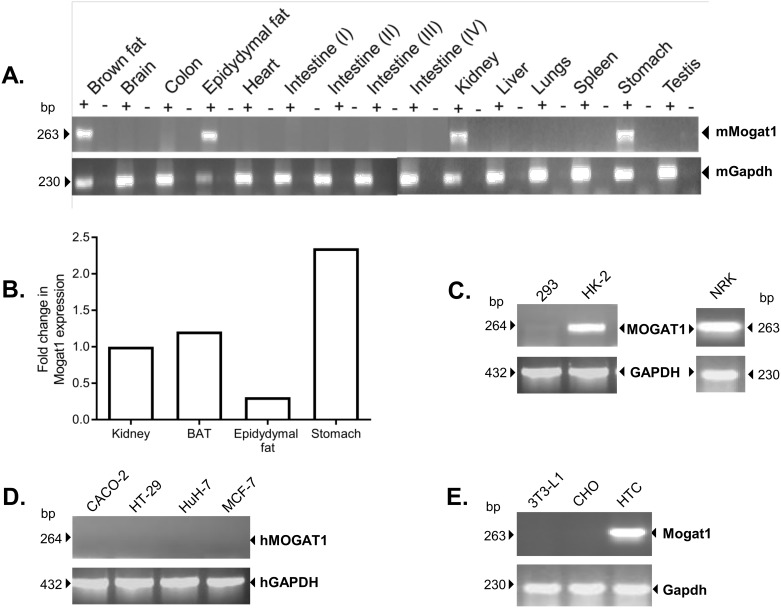
Expression of *Mogat1* in various mouse tissues and cell lines. **A.** *Mogat1* expression in several mouse tissues. *Mogat1* is expressed in brown adipose tissue, epididymal fat, kidney and stomach. **B.** *Mogat1* expression was measured by RT-qPCR in the brown adipose tissue (BAT), epidydymal fat, kidney and stomach obtained from WT mice. The data were normalized to cyclophilin and then expressed as a fold change increase in *Mogat1* compared to kidney. Stomach was found to be the highest expressing tissue. **C.** *MOGAT1* is not expressed in human embroyonic kidney (HEK-293) cells but is expressed in human proximal kidney tubule (HK-2) cells and normal rat kidney cells (NRK). **D.** Other human cell lines screened for *MOGAT1* expression: Caco-2, HT-29, Huh-7 and MCF-7. **E.** Expression of *Mogat1* in rodent cell lines: mouse fibroblasts, 3T3-L1; Chinese hamster ovary (CHO) cells; Rat hepatic tumor cells, HTC. *Mogat1* was expressed only in HTC cells. Shown is the Mogat1 product as analyzed on 1.5% agarose gels and expression of the housekeeping gene glyceradehyde phosphate dehydrogenase (Gapdh). (+) = cDNA amplification in the presences of reverse transcriptase; (-) = cDNA amplification in the absence of reverse transcriptase.

Previously, a study has shown that *Mogat1* expression is tissue restricted, being expressed in the kidney, stomach and at somewhat lower levels in the brown adipose tissue (BAT) and epididymal fat [3]. Expression of *Mogat1* in the human and mouse liver is minimally detectable [3, 18, 19]. However, it can only be detected using poly(A)^+^ RNA but not using total RNA [3]. *Mogat1* has also been reported in the livers of diet induced obese mice [6], *ob/ob* mice [7], in mouse models of diabetes like *KKAy* and *db/db* mice [5] and in cultured murine hepatocytes [20]. However, it can be appreciated that the level of *Mogat1* expression in the livers is several fold less when compared to tissues like the stomach or kidney and it is only upregulated in pathological conditions like hepatic steatosis [4]. To reconfirm the tissue distribution of mouse *Mogat1*, a conventional PCR of various mouse tissues was performed and our observations were consistent with the findings published earlier (Fig 1A). We followed this with RT-qPCR in tissues expressing *Mogat1*. Among the *Mogat1* expressing tissues we find the following order of level of expression: stomach > BAT > kidney > epidydymal fat (Fig 1B).

Though *Mogat1* is highly expressed in the stomach, there are no well-established stomach cell lines. Similarly, *Mogat1* is expressed in brown and white adipose tissue but these cells require a differentiation step rendering them unsuitable for the current study. So we were left to choose a kidney cell line as our working cellular model. We first screened various human and murine kidney cell lines which included HEK-293, HK-2 and NRK. We observed that HK-2 and NRK cells expressed *Mogat1* (Fig 1C). However, the NRK PCR product, when sequenced, did not correspond to the Genbank entry (NM_001108803) (S1 Fig). HEK-293 cells did not express *Mogat1*. In addition, we screened a few cancer cell lines which might have upregulated expression of *Mogat1* like Caco-2, HT-29, Huh-7, and MCF-7, but none of these cell lines expressed *Mogat1* (Fig 1D). Furthermore, when we amplified *Mogat1* in 3T3-L1, CHO and HTC cells, only HTC, a rat hepatoma cell line, amplified *Mogat1* which was verified by sequencing (Fig 1E). It is interesting to note that a normal mouse liver has very low levels of *Mogat1* expression whereas rat hepatoma cells express *Mogat1* at higher levels, though at this point the role of *Mogat1* in these cells is unclear. Based on these results we selected HK-2 cells as the cell culture model for studying the transcriptional regulation of the mouse *Mogat1* promoter.

### Verification of expression of *MOGAT1* open reading frame (ORF) in HK-2 cells

**Fig 2:**
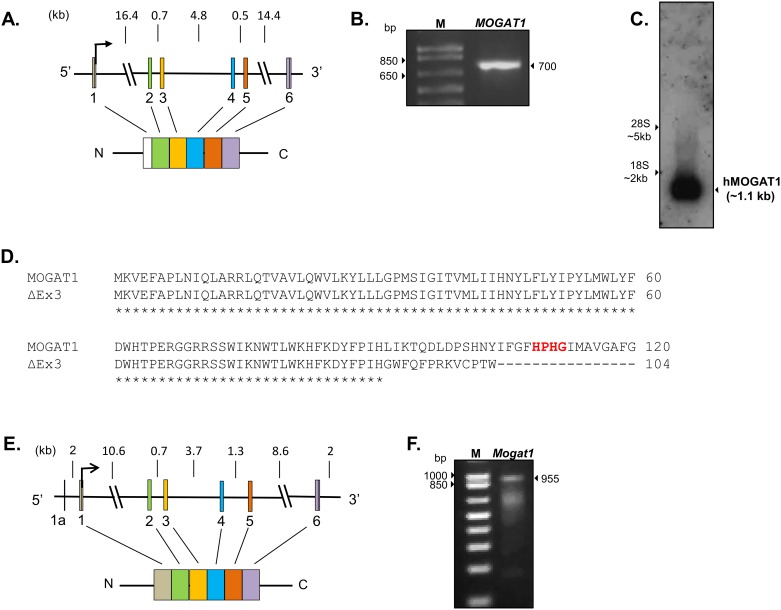
Schematic of human and mouse *MOGAT1* gene and product size. **A**. The human *MOGAT1* gene consists of 6 exons, marked as boxes. Shown below is the predicted MGAT protein. **B.** Amplification of human *MOGAT1* transcript from HK-2 cells produce a product of ~700 bp and upon sequencing confirmed the deletion of exon 3. **C.** Northern blot analysis of total RNA from HK-2 cells probed with human *MOGAT1* showing the expected size of mRNA. Northern analysis confirmed the presence of a single *MOGAT1* transcript in HK-2 cells. **D.** Amino acid alignment of the predicted MOGAT1 protein with truncated (exon 3 deleted) MOGAT1 protein in human kidney confirmed by sanger sequencing. This will result in a frame shift producing a truncated protein of p.Leu91Glyfs*13 and deletion of the putative catalytic site HPHG, marked with an asterisk. **E.** Schematic of mouse *Mogat1* gene showing exons marked as boxes. Shown below is the predicted MGAT protein. Mouse *Mogat1* has an additional untranslated exon shown as 1a. **F.** Amplification of mouse *Mogat1* mRNA of expected size and upon sequencing confirmed the predicted sequence.

Next we examined whether HK-2 cells expressed the full length ORF of *MOGAT1* transcript. The predicted ORF is 1090 bp long (GenBank accession # NC_000002) encoded from 6 exons (Fig 2A). We amplified mRNA obtained from HK-2 cells using gene specific primers designed to amplify the entire ORF (primer pairs 1_F and 2_R, Table 1). Surprisingly, the primers amplified a truncated product which was ~700 bp (Fig 2B). Upon sequencing, we observed that exon 3 was deleted. This exon contains the conserved motif “HPHG” which is the putative catalytic site for MOGAT1 enzyme (Fig 2D), suggesting that *MOGAT1* was alternatively spliced in HK-2 cells. This would result in a frameshift and an aberrant protein (p.Leu91Glyfs*13) (Fig 2D). We further confirmed this by northern blot analysis in HK-2 cells which revealed a single band of the expected size (~1.1 kb), confirming that HK-2 cells only express a single but alternatively spliced transcript of *MOGAT1* (Fig 2C) and lack a full length transcript. We further verified this observation in human kidney samples. Similar to the HK-2 cells we observed that human kidney also expresses only exon 3 deleted *MOGAT1* which was confirmed by sequencing (Fig 2D).

Based on these observations, we then verified whether mouse kidney also expresses an alternatively spliced *Mogat1*. Mouse *Mogat1* is also encoded by 6 exons (Fig 2E). Primers were designed to amplify the full length ORF including 5’UTR (Table 1). The expected PCR product of 955 bp **(**Fig 2F) was further verified by sequencing. This confirms that mouse kidney expresses an mRNA encoding the full length protein. This observation shows that there is a tissue (kidney) specific alternative splicing of *Mogat1*.

### Determination of the transcriptional start site (TSS) of *Mogat1* in HK-2 cells and mouse kidney

**Fig 3:**
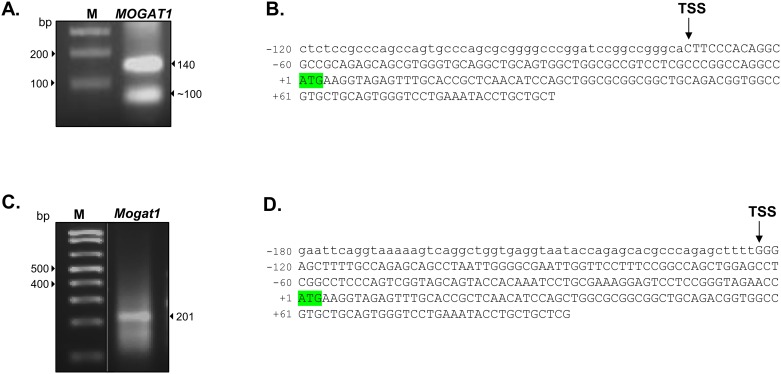
Localization of transcriptional start site (TSS) of *Mogat1* in human and mouse kidney. **A.** 5’RACE (5’ rapid amplification of cDNA ends) in HK-2 cells yielded a ~140 bp product which, upon sequencing, determined the TSS as shown in B. **B.** Shown is the TSS in relation to the translational start site (ATG) highlighted in green. **C**. Localization of TSS of *Mogat1* in mouse kidney by 5’RACE which yielded a ~200 bp product and was confirmed by sequencing. **D.** Shown is the TSS in relation to the translational start site (ATG) highlighted in green.

To define the TSS of *MOGAT1* in HK-2 cells, we performed 5’RACE. Two PCR products of ~140 bp and ~100 bp were gel purified and sequenced (Fig 3A). The ~140 bp fragment revealed the putative TSS and untranslated region whereas the ~100 bp product amplified an unrelated sequence. Thus, in HK-2 cells *MOGAT1* has one TSS which is located -72 bp from the ATG (Fig 3B). On inspection we detected no canonical TATA-box sequence (TATAAA) in the *MOGAT1* promoter suggesting that *MOGAT1* is a TATA-less promoter [21]. Most TATA-less promoters have an initiator element (Inr) for transcriptional initiation. The consensus Inr sequence (YYANWYY (C/T C/T A N A/T C/T C/T)) is also not well defined in this promoter [22]. The lack of a TATA-box or a well-defined Inr in the promoter of *MOGAT1* prompted us to search for additional elements involved in initiation of transcription. A few promoters use a downstream promoter element (DPE; RGWYVT (A/G) G (A/T) (C/T) G/A/C (T)) usually located ~28–32 bp downstream of the ATG for initiation of transcription [22]. Although not perfect, a DPE sequence, GGT, was located ~29 bp downstream from the ATG. Apart from these elements, CpG islands have been shown to be involved in transcriptional inititation [23]. Using an online bioinformatics tool, Sequence Manipulation Suite [12], for CpG island prediction, we defined the CpG island for MOGAT1 which was located from -447 to -142 bp from the ATG.

Similarly, we also defined the TSS in the mouse *Mogat1* using mouse kidney (Fig 3C). The expected PCR product was ~200 bp which was verified by sequencing (Fig 3D). In the mouse kidney the TSS was located at –123 bp from the ATG. Similar to the human *MOGAT1* promoter, the mouse promoter also lacks TATA-box and Inr sequences but had a DPE at +29 bp (GGT). The CpG island for mouse Mogat1 was located -156 to +87 bp from the ATG.

### Mapping of the proximal mouse *Mogat1* Promoter region

**Fig 4:**
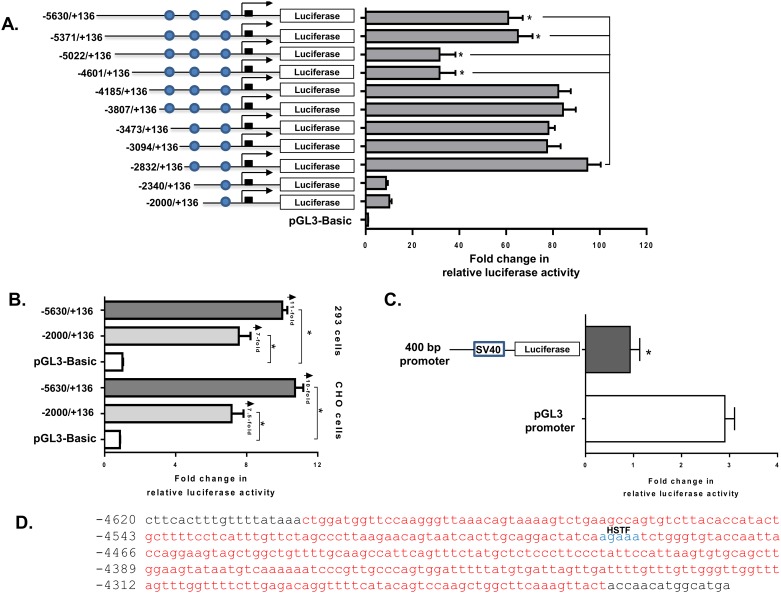
Promoter analysis of mouse *Mogat1*. **A**. Transient transfection of HK-2 cells with various serial deletion *Mogat1* promoter constructs fused to the luciferase reporter gene. The -2832/+136 construct showed a robust increase in the promoter activity (~90-fold increase). **B.** HEK-293 and CHO cells were transfected with -2000/+136 and -5630/+136 *Mogat1* promoter constructs containing the PPRE sites. Marked also are the fold chages observed compared to the empty vector, pGL3 Basic. Bars represent the fold change in relative luciferase activity. Shown are the mean±SEM from three independent experiments performed in duplicate. **C.** This region silences/reduces (2-fold) the promoter activity when inserted 5’ to the SV40 promoter Bars represent mean±SEM, n = 4, performed in duplicate. Filled circles represent the putative peroxisome proliferator response element PPRE sites. **D.** The nucleotide sequence of the ~0.5 kb region located between -4601 and -4185 which showed a -50-fold decrease in luciferase activity as shown in panel A. Shown also is predicted heat-shock transcription factor (HSTF) in blue.

Usually, the proximal promoter of a gene consists of a core promoter of ~50 bp located around the TSS and another 1 to 2 kb sequence which drives the expression of the gene and most likely includes all the genetic information required for its transcriptional activation. Reporter constructs of serial deletions of the *Mogat1* promoter region were generated from the ~5.5 kb sequence upstream from the ATG. Luciferase activity was measured in transiently transfected HK-2 cells. The strongest luciferase activity (~94-fold) was recovered with the construct containing the -2832/+136 region (Fig 4A). The luciferase reporter activity in the presence of additional 5’ sequences resulted in no additional increase in the reporter gene activity: Mogat1-3094, Mogat1-3473, Mogat1-3807 and Mogat1-4185 plasmids ~77-, ~78-, ~84- and ~82-fold respectively (not statistically significant compared to -2832/+136) (Fig 4A). This suggests that these regions do not contain any additional genetic information for the transcriptional regulation of *Mogat1*. Interestingly, when an additional promoter region from -4185 to -5022 was included we observed a ~50-fold decrease (compared to -2832/+136, p≤0.05) in the luciferase activity suggesting the presence of a repressing element in this region (Fig 4A). Next, when we included more of the 5’-region from -5022 to -5630, this repression of the *Mogat1* transcription was partially overcome (~33-fold increase compared to -4185/+136, p≤0.05). Thus, these observations suggests that the -2832 upstream region may be the most critical region in the *Mogat1* promoter which might include the proximal promoter along with the primary regulatory elements.

To define if the above *Mogat1* promoter sequences are sufficient to drive its transcription in non-permissive cells like HEK-293 and CHO cells, we also measured the luciferase-reporter activity in these cell types. We tested constructs consisting of -2000/+136 and -5630/+136 of the Mogat1 promoter. Both the constructs resulted in ~7 to ~11-fold increase in the luciferase-reporter activity (Fig 4B), suggesting that the tissue specific regulatory elements are located outside of this promoter region.

### Presence of a repressor element in ~4 kb upstream region of mouse *Mogat1* promoter

As described above (Fig 4A), we noted a decrease in mouse *Mogat1* promoter activity due to the presence of the sequences -4185 to -4601 bp and -4601 to -5022 bp. Since both the reporter constructs have similar luciferase activity, the silencer/repressor elements are more likely located within the -4185 to -4601 bp region. To test the silencer/repressor effect of this region a 400 bp fragment from -4185 to -4601 was cloned in front of the SV40 promoter (pGL3-promoter vector). As shown in Fig 4C, the transfection of HK-2 cells with this plasmid resulted in a ~50% decrease in luciferase activity compared to the pGL3-promoter, thus confirming the presence of a silencer repressor element. We found a heat shock transcription factor (HSTF) binding site in this region shown in blue (Fig 4D) [24]. Heat shock protein has been shown to act as a silencer in the transcription of interleukin-1 [25], although it is unclear from this study if HSTF is the main suppressor element.

### Conserved regulatory elements in the promoters of mouse and human Mogat1

**Fig 5:**
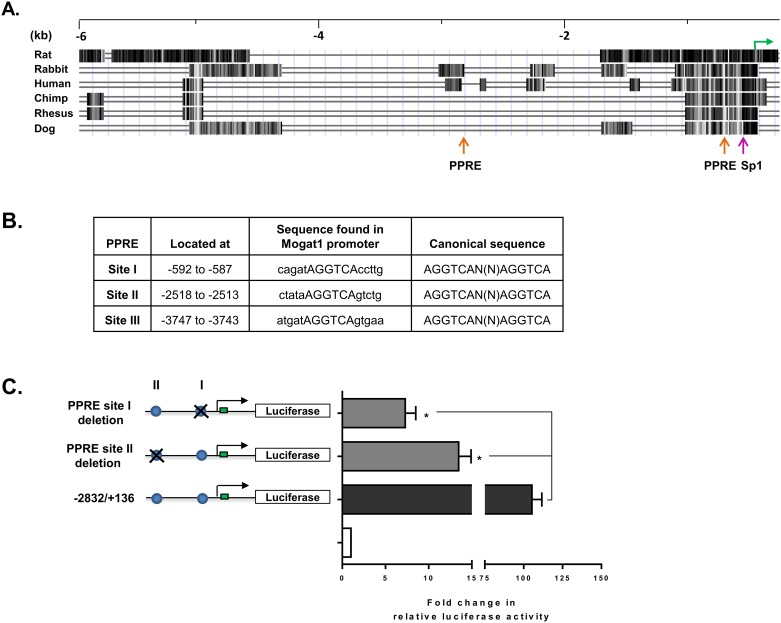
Deletion of peroxisome proliferator response element (PPRE) sites in mouse *Mogat1* promoter reduces its transcriptional activity. **A.** Mogat1 multigenome alignment from exon 1 to ~6 kb upstream. Orange arrows represent PPRE sites and the green arrow represents ATG. **B.** Shown are the locations of the PPRE sites and sequences found in the mouse promoter along with the canonical PPRE sequence. Note in the mouse promoter only half the PPRE site is found. **C.** PPRE deleted *Mogat1* promoter analysis in HK-2 cells. The PPRE sites I and II were deleted in the -2832/+136 Mogat1 promoter construct. Deletion of site I decreased the luciferase activity to ~7.5-fold and site II to ~15-fold. Bars represent the mean±SEM (n = 3) performed in duplicate.

We next searched for the putative *cis*-acting elements in the ~5.6 kb region of the mouse Mogat1 promoter to generate a lead transcription factor(s) (TF) which might regulate the Mogat1 transcriptional activity. Employing bioinformatics tools like TRANSFAC (TRANScription FACtor database) and UCSC genome browser, we determined the conserved regions among various species and identified putative TF sites (Fig 5A). A few TFs like specificity protein (Sp1) and peroxisome proliferator-activated receptor (PPAR) are noteworthy. In TATA-less promoters, Sp1 is an important TF regulating transcriptional activation. PPARs are ligand activated nuclear receptors regulating transcriptional activation of several genes. While these sites are located in the conserved region of all three species, there are additional sites which fall outside these conserved regions. Peroxisome proliferator-activated receptor response elements (PPRE) were located at -2518 and -3747 kb (Fig 5B). Though other *cis*-regulatory elements were also predicted in the *Mogat1* promoter (S2 Table), in this study we focused on PPAR, which is known to be involved in hepatic steatosis and lipid accumulation [26].

The luciferase reporter gene assay of the promoter construct Mogat1–2832/+136 bp of the mouse Mogat1 showed strongly enhanced luciferase activity and also carries the two predicted PPRE sites as shown in Fig 5B. To confirm the function of these PPRE binding sites, we deleted these sites individually in the Mogat1–2832/+136 bp construct and measured the luciferase-reporter activity in HK-2 cells. The deletion constructs Mogat1-del-PPRE-site1-2832/+136, and Mogat1-del-PPRE-site2-2832/+136 both reduced the transcriptional activity when compared to those of undeleted constructs (Fig 5C). PPRE-site 1 deletion (del-1) resulted in an ~85% decrease (p value = 0.003) and PPRE-site 2 deletion (del-2) led to a ~90% reduction (p value = 0.003) in the Mogat1 promoter activity. Based on the ~85–90% decrease in Mogat1 promoter activity, we deemed it unnecessary to delete both the PPRE site as well as to study the third PPRE site located further upstream (-3747 bp).

### Specificity of PPRE site located on mouse Mogat1 promoter

**Fig 6:**
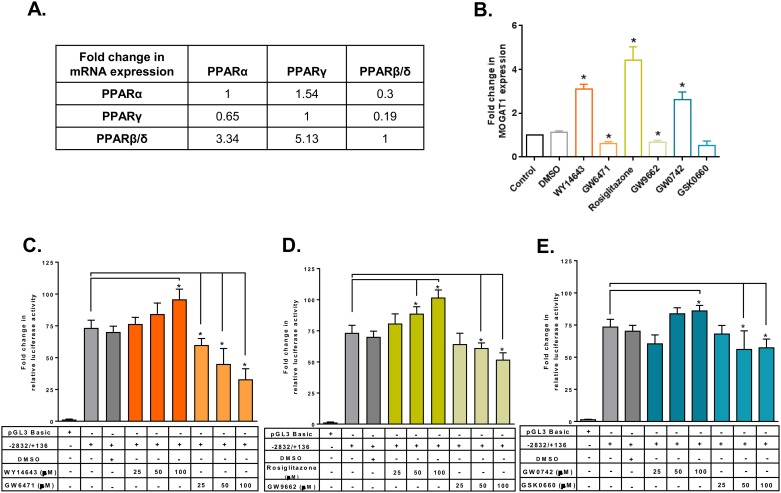
PPAR agonists and antagonists regulate the expression of *Mogat1* in HK-2 cells. **A.** RT-qPCR analysis of PPAR isoforms expression in HK-2 cells. All the PPAR isoforms are compared with each other. PPARβ/δ > PPARγ > PPARα. **B. **RT-qPCR analysis of *MOGAT1* expression in HK-2 cells treated with various agonists and antagonists of PPAR isoforms. Bars represent mean±SEM (n = 4) performed in duplicate. (*) p value ≤0.05. **C.** Changes in the relative luciferase activity of the mouse *Mogat1* promoter (-2832/+136) consisting of both the PPRE sites in the presence of PPARα agonist WY16463 and antagonist GW6471. Shown are the fold changes compared to the pGL3-basic construct and normalized to protein. **D.** Fold change in relative luciferase activity in the presence of PPARγ agonist rosiglitazone and antagonist GW9662 and normalized to protein. **E.** Fold change in relative luciferase activity in the presence of PPARβ/δ agonist GW0742 and antagonist GSK0660 and normalized to protein. Bars represent mean±SEM (n = 4) performed in duplicate. (*) p value ≤ 0.05.

PPARs are ligand activated TFs which belong to the superfamily of nuclear receptors. PPARs can function as homodimers or form heterodimers with retinoid X receptor (RXR). PPARs bind to PPRE composed of a direct repeat (DR) preferably spaced by one nucleotide (DR1) with a consensus sequence of AGGTCA-N-AGGTCA. PPARs also recognize DR2 motifs that are preferably spaced by two nucleotides [27, 28]. We first measured the expression pattern of PPARs in the HK-2 cells and found that the highest expressing isoform was PPARβ/δ followed by PPARα and PPARγ (Fig 6A). We then measured the expression of human *MOGAT1* in the presence of agonists and antagonists for all three PPAR isoforms. *MOGAT1* expression was increased ~3-, 4- and 2.5-fold in the presence of PPARα, γ, and β/δ agonists WY14643, rosiglitazone and GW0742, respectively (Fig 6B). On the other hand, in the presence of PPARα, γ, and β/δ antagonists GW6471, GW9662 and GSK0660, *MOGAT1* expression was inhibited ~0.6-, 0.7- and 0.6-fold, respectively. We further verified this by measuring the activation of the mouse Mogat1 promoter luciferase- reporter construct (-2832/+136 bp) in the presence of the PPAR agonists and antagonists. Remarkably, we observed that all three PPAR agonists activated the promoter to the same level. In the presence of 25, 50 and 100 μM of PPARα agonist WY14643, 3-, 11- and 22-fold increase in the luciferase reporter activity was observed. Similarly, a dose-dependent response was observed in the presence of PPARγ agonist rosiglitazone (7-, 15-, 28-fold) and PPARβ/δ agonist GW0741 showed 10-12-fold increase at 50 and 100 μM doses, although at a lower dose of 25 μM it has no effect (Fig 6C–6E). At the highest dose of 100 μM of PPAR agonist we observed that the increase in luciferase reporter activity was statistically significant. The antagonist activity of PPARα was also dose-dependent, inhibiting the luciferase reporter activity with progressive escalation of the antagonist GW6471 (13, 28, 40-fold decrease). The other PPAR isoforms resulted in a modest decrease in the luciferase reporter activity: ~9-, 12-, 21- fold decrease for PPARγ and ~5-, 17-, 16-fold decrease for PPARβ/δ. At the highest dose of 100 μM of PPAR antagonist we observed that the inhibition of luciferase reporter activity was statistically significant. This observation did not provide evidence for the specificity of the PPARs.

**Fig 7:**
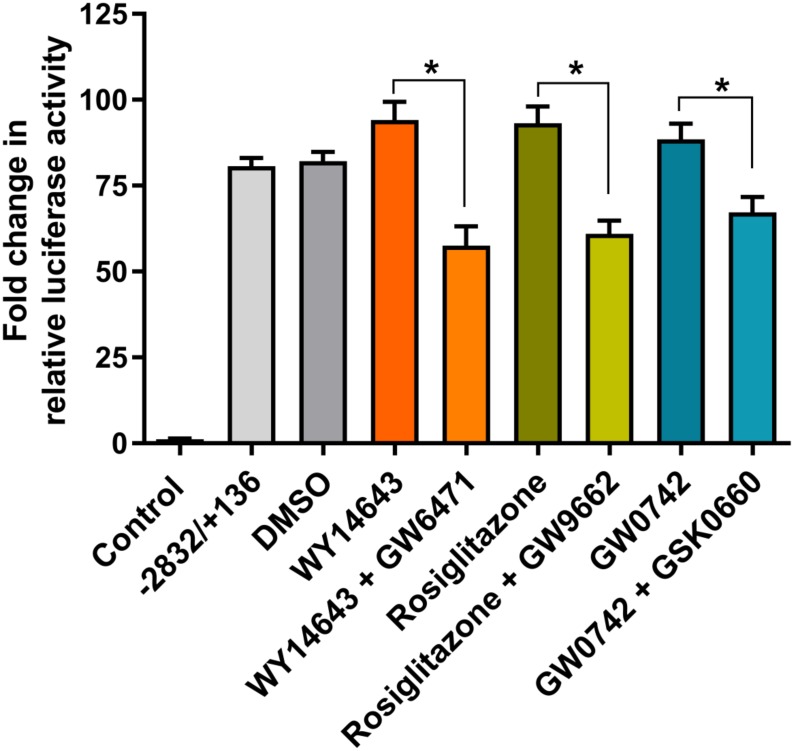
Specificity of PPAR agonists in HK-2 cells. Changes in the relative luciferase activity of the mouse *Mogat1* promoter (-2832/+136) consisting of both the PPRE sites in the presence of PPARα agonist WY16463 and its antagonist GW6471; PPARγ agonist rosiglitazone and its antagonist GW9662; and PPARβ/δ agonist GW0742 and its antagonist GSK0660. Shown are the fold changes compared to the pGL3-basic construct and normalized to protein. Bars represent mean±SEM (n = 3–4) performed in duplicate. *p value ≤ 0.05 shows the statistical significance between PPAR specific agonist and antagonist.

To further determine the specificity of each of the PPAR agonists, we performed a competition experiment using a 100 μM agonist and an equimolar concentration of antagonist. The mouse *Mogat1* promoter luciferase-reporter construct (-2832/+136 bp) was activated by PPARα agonist WY14643. This activation was inhibited ~34-fold in the presence of PPAR antagonist GW6471 (Fig 7). Similarly, *Mogat1* promoter activation by the PPARγ agonist rosiglitazone was inhibited ~32-fold by its antagonist GW9662. The activation of *Mogat1* promoter by GW0742 was inhibited by ~20-fold in the presence of its antagonist, GSK0660. The competition between PPARβ/δ agonist and antagonist was not as strong as those observed for PPARα and PPARγ. This further shows the specificity of each of the PPAR agonist used in this study.

### Fatty acids and phosphatidic acids do not activate *Mogat1* in primary mouse hepatocytes

Fatty acids and fatty acid derived molecules have been shown to activate PPARs [29]. Fatty acids for this study were chosen based on the findings from Xu et al. [30]. Primary mouse hepatocytes isolated from the livers of WT mice were incubated with BSA-conjugated fatty acids (300μM). *Mogat1* expression remained unchanged in the presence of all fatty acids tested: the saturated fatty acids palmitic acid (C16:0) and stearic acid (C18:0), monounsaturated oleic acid (C18:1), and polyunsaturated arachidonic acid (C20:4) (S2 Fig).

We have previously observed that PA induced the expression of gluconeogenic genes in primary mouse hepatocytes [15]. We measured *Mogat1* expression in the presence of PA species which have been shown to increase gluconeogenesis in primary hepatocytes of *Agpat2*^*-/-*^ mice. *Mogat1* expression in *Agpat2*^*-/-*^ hepatocytes remained unchanged in the presence of C16:0/18:1 PA (~0.2 fold decrease). This suggests that PA did not have any role in the transcriptional regulation of *Mogat1* expression.

### ChIP assay confirms occupation of PPRE site in *Mogat1* promoter by PPARα

**Fig 8:**
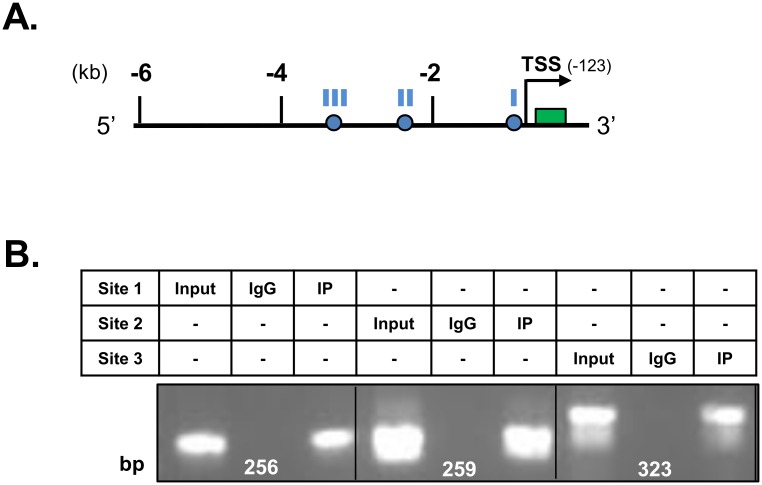
Chromatin immunoprecipation (ChIP) assay for binding of peroxisome proliferator response element (PPRE) on *Mogat1* promoter. **A**. Schematic of mouse *Mogat1* promoter showing the three PPRE sites located at -592 (site I), -2518 (site II) and -3747 (site III) bp in relation to ATG. Circles represent PPRE sites, the green box is exon 1 and the arrow head represents the TSS. **B**. Mouse kidney homogenate was immunoprecipitated with PPARα antibody and the PPRE sites were amplified with respective primer pairs. The immunoprepcipitated DNA fragment was sequenced and contained the desired nucleotide sequences. All 3 PPRE sites are occupied by PPARα. Shown is the representative image from replicate experiments (n = 2).

We have shown above that PPARα regulates *Mogat1* transcription. To further validate whether the mouse *Mogat1* PPRE sites are occupied by PPARα, we performed chromatin immunoprecipitation (ChIP) assays. The mouse kidney homogenate was immunoprecipitated with anti-IgG and anti-PPARα antibodies and DNA was amplified. Although all PPRE sites were occupied by PPARα (Fig 8A and 8B), it seems that the proximal two PPRE sites are sufficient to regulate *Mogat1* promoter by its ligand.

**Fig 9:**
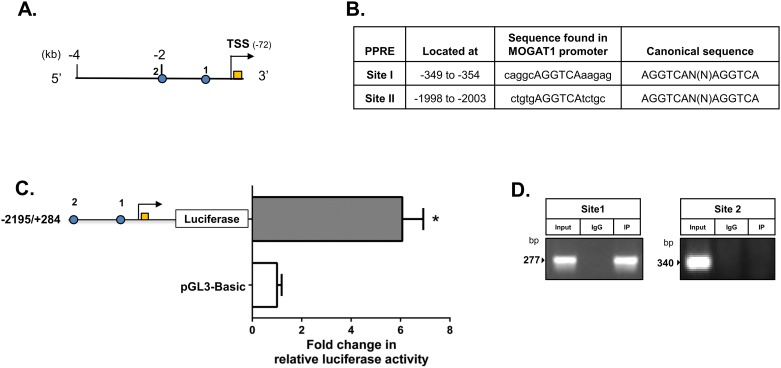
Chromatin immunoprecipation (ChIP) assay for binding of peroxisome proliferator response element (PPRE) located in *MOGAT1* promoter. A. Schematic of human *MOGAT1* promoter showing the two PPRE sites located at -349 (site 1) and -1998 (site 2) bp in relation to ATG. Circles represent PPRE sites, the orange box is exon 1 and the arrow head represents the transcriptional start site (TSS). B. Shown are the locations and sequences of the PPRE sites found in the human promoter. C. Transient transfection of human *MOGAT1* promoter construct in HK-2 cells. Bars represent the fold changes in relative luciferase activity shown as mean±SEM (n = 3) performed in duplicate. D. ChIP assay in cell lysate from HK-2 cells probed with PPARα antibody and the amplified product analyzed on 1.5% agrose gel. Among the two PPRE sites only site 1 located at -349 was occupied by PPARα. Shown is the representative image from replicate experiments (n = 2).

Bioinformatics also predicted two PPRE sites in the ~2 kb upstream region of human *MOGAT1* promoter. The two PPRE sites are located at -349 and -1998 bp (Fig 9A and 9B). Several other predicted TF binding sites are shown in Table 2. To determine if these PPRE sites regulate the *MOGAT1* promoter, we generated a promoter luciferase-reporter construct consisting of the -2195/+284 region. HK-2 cells transfected with this construct showed a 6-fold increase in luciferase activity (Fig 9C). ChIP assay confirmed that only one site (present at -349) is occupied by PPARα (Fig 9D).

**Table 2:** Predicted transcription factors (TFs) in ~20 kb region of human *MOGAT1* promoter. The putative TF binding sites were identified by bioinformatics tools, TESS and TRANSFAC. Shown are only few of the transcription factors present in regions A to F.

TFBS	Region A	No. of sites	Region B	No. of sites	Region C	No. of sites	Region D	No. of sites	Region E	No. of sites	Region F	No. of sites	Canonical sequence
alcohol dehydrogenase regulator 1	ADR1	2	ADR1	14	ADR1	5	ADR1	13	ADR1	7	ADR1	2	GGAGA
helix-loop-helix transcription factor	E12	1	E12	4	E12	1	E12	1	E12	2	E12	1	CAACTG or CAGCTG or CATCTG
Heat shock transcription factor	HSTF	12	HSTF	12	HSTF	12	HSTF	19	HSTF	18	HSTF	10	AGAAA
CCCTC-binding factor (zinc finger protein)	CTCF	15	CTCF	12	CTCF	6	CTCF	12	CTCF	1	CTCF	5	CCCTC
Yin Yang 1	YY1	1	YY1	7	YY1	5	YY1	11	YY1	30	YY1	3	ANATGG
Hepatocyte nuclear factor	HNF-3	1	HNF-3	3	HNF-3	4	HNF-3	6	HNF-3	5			GTAAATA or TATTTGT
Pituitary-specific positive transcription factor	POU1f1a	2	POU1f1a	7	POU1F1a	6	POU1F1a	9	POU1F1a	17			TAAAT
T-cell acute lymphocytic leukemia 1	Tal-1	1	Tal-1	10	Tal-1	3	Tal-1	5	TAL-1	1			CACCTG
homeobox	HOXA5	2	HOXA5	6	HOXA5	3	HOXA5	1			HOXA5	4	CTGATG
Upstream stimulatory factor 1	USF-1	1	USF-1	9	USF1	1	USF1	1			USF1	2	CACATG
Nuclear factor	NF-1/L	3	NF-1/L	8	NF-1/L	7	NF-1/L	4			NF-1/L	6	TGGCA(N5)TGCCA
Steroidogenic factor 1	SF-1	1	SF-1	2	SF-1	2	SF-1	1			SF-1	1	CCAAGGTCA
CCAAT displacement protein	CUTL1	15	CUTL1	2			CUTL1	1					CACATG
Actin-Related Protein 1	ARP-1	2	ARP-1	5									
hematopoietic transcription factor	PU.1	1	PU.1	3									TTCCTC
Ecotropic viral integration site 1	Evi-1	1	Evi-1	4									TGACAAGATAA
Peroxisome proliferator activator protein	PPAR-a	2	PPAR-a	1									AGGTCA
CCAAT/enhancer-binding protein alpha					alpha-CBF	1	alpha-CBF	1	alpha-CBF	1	alpha-CBF	1	ATTGGGCAAT
POU class 2 homeobox 1					POU2F1	1	POU2F1	1	POU2F1	3	POU2F1	3	ATTCCATTA
core-binding factor alpha					C/EBPbeta	4	C/EBPbeta	3			C/EBP	4	
hepatocyte-specific nuclear protein					APF-1	2	APF-1	1					CTGGAAA
estrogen receptor							ER	4	ER	9	ER	1	GGGGCGGGG or GGTGTGGGG
Specific protein							SP-1	1	SP-1	8			GGTCANNNTGACC
Activator protein 1							AP-1	3					TGA[G/C]TCA
homeobox-containing transcription factor							Nkx2-5	1					TYAAGTG

### Chromosome conformation capture (3C) assay in HK-2 cells confirms long range DNA looping interactions of human *MOGAT1* promoter

The above study of the *Mogat1* proximal promoter reveals basic *cis*-regulatory elements for its transcriptional activation, but not in the context of a whole genome. The functional connectivity of genes and regulatory elements can be mapped by identification of physical interactions between them. To analyze the frequency of interaction and proximity between any two genomic loci and their impact on gene expression, Dekker and colleagues developed the 3C assay [31]. This method pulls down the long range interacting complexes and helps to further reveal the *cis*-interacting elements which come in close proximity to each other in the presence of enhancers or suppressors. The principle behind this method is to crosslink the *trans*-interacting protein(s) to the chromatin DNA in the intact nuclei. The protein-DNA complexes are digested and ligated for capturing the intra- or inter-molecular interactions. The ligated DNA is reverse-crosslinked and amplified by PCR using specific primer sets to identify the interacting genomic region.

**Fig 10:**
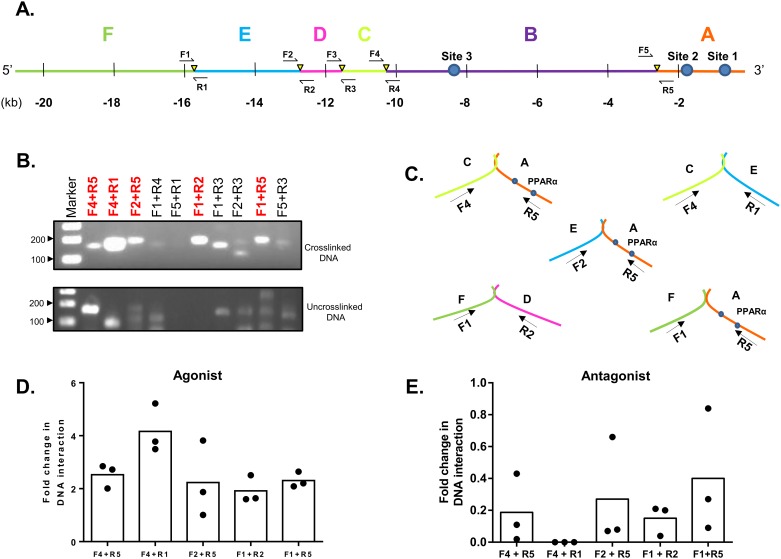
Chromosome conformation capture (3C) assay of human *MOGAT1* in HK-2 cells. **A.** Schematic of the ~20 kb region of the *MOGAT1* promoter drawn to scale showing BglII sites (yellow triangles) and PPRE sites (filled circles). The DNA fragments formed after BglII digestion are marked as A, B, C, D and E. **B.** DNA fragments from crosslinked and un-crosslinked samples were amplified using primer pairs shown in panel A and analyzed on a 2% agarose gel. **C.** Schematic for various DNA-DNA interactions which were further confirmed by sequencing. **D-E.** RT-qPCR analysis of the five DNA-DNA interactions in the presence of WY16463 (PPARα agonist) and GW6471 (PPARα antagonist). Dots represent individual experiments and bars represent the mean from three independent experiments.

We initially attempted the 3C assay in the mouse whole kidney and kidney primary cells. Despite our efforts, we could not get the 3C assay to work reliably and it will require further optimization for detecting the DNA-DNA interactions in the mouse kidney. However, we were successful in detecting the long range genomic interactions using HK-2 cells, which is our chosen cell culture model. Various combinations of primer sets were used to amplify the possible DNA interacting regions (Fig 10A). The PCR product obtained with primer pairs F4 and R5 confirmed an interaction between regions C and A of the *MOGAT1* promoter (Fig 10B). Similarly PCR products with primer pairs F2 and R5 confirmed an interaction between regions E and A (Fig 10B**)**. Genomic region F interacts with two regions: A and D. Similarly, region A, which contains the PPARα sites, interacts with the three regions C, E and F. All the PCR products obtained were sequenced to verify the predicted sequence. How these interactions are brought about, or the sequence in which these interactions occur, is still unclear. These interactions suggest that the PPARα site present in the region A was most active and formed more loops with the distant upstream regions compared to other PPARα sites.

These DNA-DNA interactions are responsive to PPARα agonist and antagonist. Real time PCR data demonstrated a ~2.8-fold increase in DNA-DNA interaction between regions C and A in the presence of PPARα agonist WY14643 and a negligible decrease (~0.2-fold) in the interaction in the presence of PPARα antagonist GW6471 (Fig 10D and 10E). Similarly, a ~4-fold increase in DNA-DNA interaction between regions C and E in the presence of WY14683 and a complete inhibition in the presence of GW6471 was observed. The interaction at regions E and A showed a ~2-fold increase in the presence of the agonist and a negligible decrease (0.3-fold) in the presence of the antagonist. The interaction between regions F and D was least responsive compared to other regions with only ~1.8-fold increase in the presence of agonist. DNA-DNA interaction between regions F and A showed ~2.2-fold increase in the DNA interaction in the presence of WY14643 and a negligible decrease (~0.4-fold) in the presence of antagonist. Though all the interactions showed a change in the presence of agonist and antagonist, the most effective interaction seemed to be between regions C and E suggesting that, apart from PPRE, other regulatory elements also participate in *Mogat1* transcriptional regulation.

### Study of *Mogat1* in rat hepatic tumor cells (HTC)

We also attempted to verify the expression of *Mogat1* in HTC cells. Interestingly, though *Mogat1* is minimally expressed in the adult liver, we could amplify exons 4–6 of *Mogat1* in HTC cells (Fig 1D). To confirm whether the entire open reading frame (ORF) of *Mogat1* is expressed in these cells we used additional primers located in the entire ORF. The primers were designed based on the *in silico* prediction of the rat *Mogat1* gene. Based on the “multiz align and conservation” at the UCSC genome browser we noted that human, mouse and rat *Mogat1* exon-intron boundary and gene structure is highly conserved (S3A Fig). Based on the predicted cDNA for rat *Mogat1* we were only able to amplify exons 4–6 (S3B and S3C Fig). Our repeated attempts to amplify exons 1–3 remained unsucessful. This might be due to chromosomal rearrangement in HTC cells. Nevertheless, the amplification of exons 4–6 also suggests that the promoter region for the expression of *Mogat1* is still intact. Since our studies are directed towards the analysis of the *Mogat1* promoter we believe that HTC cells could represent the liver cells and be useful in analyzing the *Mogat1* promoter.

**Fig 11:**
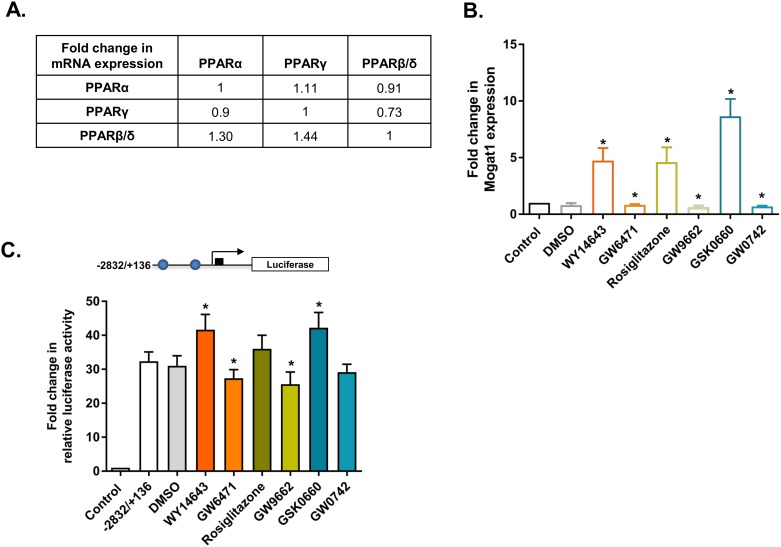
Expression of PPAR isoforms, their activation and inhibition of *Mogat1* in rat hepatic tumor (HTC) cells. **A.** RT-qPCR analysis of PPAR isoform expression in HTC cells. All the PPAR isoforms are compared with each other. PPARβ/δ > PPARα > PPARγ (n = 5). **B.** RT-qPCR analysis of *Mogat1* expression in HTC cells treated with 100μM of various agonists and antagonists of PPAR isoforms. Bars represent mean±SEM (n = 5) performed in duplicate. **C.** Changes in the relative luciferase activity of the mouse *Mogat1* promoter (-2832/+136) consisting of both the PPRE sites in the presence of PPAR agonists (100μM; PPARα-WY16463, PPARγ-rosiglitazone and PPARβ/δ-GW0742) and antagonists (100μM; PPARα-GW6471, PPARγ-GW9662, PPARβ/δ-GSK0660). Shown are the fold changes compared to the pGL3-basic construct and normalized to protein. Bars represent mean±SEM (n = 5) performed in duplicate. *p value ≤ 0.05.

The endogenous expression of PPARs were quantified by real-time PCR in the HTC cells. The expression pattern of PPARs in the HTC cells are different than those of HK-2 cells. The highest expressing isoform was PPARβ/δ followed by PPARα and PPARγ (Fig 11A). The predicted PPREs are highly conserved amongst the species as shown in Fig 5A. To measure whether these PPARs are biologically active in HTC cells we measured the activation of *Mogat1* in the presence of agonists and antagonists for all three PPAR isoforms. *Mogat1* expression was increased ~5-, 5- and 8-fold in the presence of agonist (100 μM) for PPARα (WY14643), PPARγ (rosiglitazone) and PPAR β/δ (GW0742) (Fig 11B). On the other hand, in the presence of antagonist (100 μM) for PPARα (GW6471), PPARγ (GW9662) and PPARβ/δ (GSK0660) *Mogat1* expression was inhibited ~0.8-, 0.8- and 0.6-fold, respectively. Except for the inhibition of *Mogat1* by PPARγ (GW9662), none reached statistical significance (Fig 11B). To verify if these PPARs could activate the promoter we measured the activation of the mouse *Mogat1* promoter luciferase-reporter construct (-2832/+136 bp) in the presence or absence of PPAR agonists and antagonists. Interestingly, we observed that only PPARα and PPARβ/δ agonists activated the promoter while PPARγ agonist had no effect. In the presence of PPARα agonist (WY14643) and PPARβ/δ agonist (GW0742) a ~9- and ~10-fold increase in the luciferase reporter gene was observed, respectively (Fig 11C). On the other hand, while PPARγ agonist (rosiglitazone) had no effect on the luciferase reporter gene, PPARγ antagonist, GW9662, was the most effective in inhibiting the expression of *Mogat1* (~7-fold decrease).

We then attempted the 3C assay in HTC cells as performed in HK-2 cells. In the rat *Mogat1* promoter we found that *HindIII* restriction sites were evenly distributed and could reasonably cross-link DNA-DNA interactions. Primer pairs were designed across a ~20 kb region of rat *Mogat1* promoter and amplified the possible DNA-DNA interacting regions (S4A Fig). All the primer pairs could amplify the DNA regions in the uncross-linked control genomic DNA (S4B Fig) which were confirmed by sequencing. However, we were unsuccessful in amplifying any DNA-DNA interacting regions as we saw in the HK-2 cells. The only DNA interaction we observed was with primers F2 and R6 located in regions ‘I’ and ‘D’ of the rat *Mogat1* promoter (S4A Fig). Though the amplified product shown in S4C Fig was of expected size, only the region downstream from primer F2 was verifiable by sequencing but not from R6 primer. Upon nucleotide homology search (NCBI BLAST program), the sequences amplified by R6 primer did not match the expected sequences.

## Discussion

In this study we provide an extensive and detailed characterization of the mouse and human Mogat1 promoter in kidney and hepatic cell lines. We show that both mouse and human *MOGAT1* promoters lack TATA-box and initiator (Inr) sequences or a downstream core promoter element (DPE) [22, 32]. However, promoters for both the species have CpG island (CGI). GC-rich regions are found in the proximal regions of several promoters which are shown to initiate transcription [23].

Human kidney expresses a truncated MOGAT1 transcript (this study) but this is not the case in the mouse kidney. Evolutionary conservation of genes shows that only those genes, and by extension proteins, which are functionally required in animals’ physiology survive. It is unclear why it is expressed in the mouse kidney as a functionally active protein, while in the human kidney it is alternatively spliced to generate a catalytically inactive enzyme. It could be speculated that during evolution the expression of catalytically active Mogat1 protein interfered with human kidney physiology and was inactivated by an alternative splicing mechanism. Similar to the human kidney, the human liver also expresses the alternatively spliced MOGAT1 transcript [19]. The importance of alternative splicing of transcripts in mammalian physiology which results in pathological conditions, including cancer, is well established [33]. To further understand the species and tissue-specific alternative splicing of MOGAT1, a greater knowledge of the molecular mechanisms involved in its alternative splicing is required.

While our goal was to understand the transcriptional regulation of *Mogat1* to devise additional ways to suppress hepatic TAG synthesis, targeted genetic deletion of *Mogat1* in *Agpat2*^*-/-*^ and *ob/ob* genetic backgrounds did not attenuate the hepatic TAG [8]. It should also be noted that *Mogat1* is highly expressed in tissues like the kidney and stomach which are not known to synthesize any significant levels of neutral lipids such as DAG or TAG. The function of *Mogat1* in these tissues is still unclear. *Mogat1* can synthesize DAG from MAG. DAG is a signaling molecule on its own and is also a precursor for phospholipid synthesis. Phospholipids are critical lipids both as signaling molecules and by providing a structural backbone for cellular membranes. From this perspective, the characterization of the *Mogat1* promoter (the current study) will provide a lead to study its role in these tissues. Although we were unable to show a robust decrease in liver TAG or improved glucose tolerance in the *Mogat1*^*-/-*^*;Agpat2*^*-/-*^ and *Mogat1*^*-/-*^*;ob/ob* mice [8], it might still be possible to further understand the transcriptional regulation of *Mogat1* in other tissues like stomach and kidney.

We also show that PPARα agonist and antagonist also modulate the ~2.8 kb mouse *Mogat1* promoter activity when expressed in HK-2 cells. This suggests that PPARα also modulates *Mogat1* promoter activity (this study), in addition to PPARγ which also occupies the PPRE site [30]. Similarly, the human *MOGAT1* promoter also contains PPRE sites which are occupied by PPARα as determined by ChIP assay. Two recent reports [9, 10] also show that PPARγ directly binds to the PPRE in the mouse and human *Mogat1* proximal promoter and activates its expression. These experiments were conducted in 293T, HepG2 cells and primary human hepatocytes. From these studies it is also unclear whether PPARγ is the only receptor that could activate *MOGAT1*. Reduction in the levels of liver TAG and expression of liver *Mogat1* have been achieved both by using antisense oligonucleotide (ASO) inhibition in obese mice and were also observed in a liver specific PPARγ knockout mouse on a high fat diet [6]. On the other hand, we show that PPRE in mouse *Mogat1* is capable of responding to all three PPARs (using agonists and antagonists) and also that PPARα specifically occupies the putative PPRE site *in-vivo* (ChIP assay).

In hepatic steatosis, expression of several genes is dysregulated, including PPARγ and PPARα. While PPARγ has been studied previously [9, 10], from the clinical perspective PPARγ agonist might not be the best therapeutic agent due to the risk of developing heart failure [34]. PPARγ antagonist also might not be beneficial because it will interfere with adipogenesis. Therefore, we focused our study on characterizing the interaction of *Mogat1* with PPARα. PPARα increases hepatic fatty acid oxidation and prevents hepatic steatosis [35]. *Pparα*^*-/-*^ mice display increased hepatic steatosis, oxidative stress, and inflammation when fed a high fat diet [36, 37] but not on normal chow. Thus, inhibiting its activity in a clinical setting might not yield the desired results of reducing hepatic fat. Therefore, inhibiting either PPAR isoform will not be beneficial. Futher studies are required to explore the role of PPARβ/δ reported in this study.

The most interesting observation of this study is the long range DNA-DNA interaction which brings together upstream *cis*-elements to regulate transcriptional activation of *Mogat1*. Our 3C assay shows at least five different DNA-DNA interactions where the DNA region A, (DNA fragments named A-E for convenience, Fig 10A), which has the PPARα sites, interacts with regions C, E and F. However, region B, which also harbors a PPRE site, showed no interaction with any of the flanking regions and thus this PPRE site most likely did not contribute towards *Mogat1* transcriptional activiation in the context of the whole genome. In the presence of PPARα agonist, interaction between regions C and E was enhanced and conversely, this interaction was almost undetectable with PPARα antagonist (Fig 10D and 10E). This reveals that the regions C and E interact with A and thus regulate the expression of *Mogat1*.

**Fig 12:**
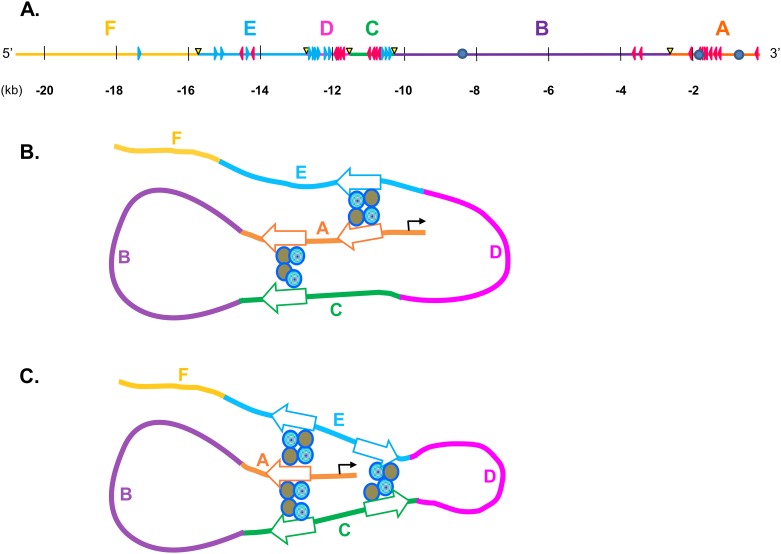
Predicted CCCTC-binding factor (CTCF) sites in the ~20 kb region of human *MOGAT1* promoter and possible interaction. **A.** *MOGAT1* promoter drawn to scale showing BglII sites (yellow triangles). The orientation of CTCF sites are shown as they occur in the promoter. Blue triangles represent sites present in forward orientation and red triangles represent sites present in reverse orientation. **B-C.** Speculative schematic for DNA-DNA interactions as determined by chromosome conformation capture (3C) assay. CTCF sites are shown to interact with each other in opposite orientation and require cohesin protein, shown as circles. The arrow heads show the orientation of CTCF sites. All regions are color coded and labeled A-F for clarity.

*In silico* analysis of the *MOGAT1* 5’-regulatory region predicts several TFs (Table 2). The most interesting seems to be CCCTC-binding factor (CTCF) [38, 39]. CTCF has been shown to play an important role as a modulator of transcription by forming DNA loops between promoters and enhancers [39]. DNA interactions occur when the CTCF *cis*-elements are in opposite orientation [40]. Thus, based on the predicted CTCF sites and orientation (Fig 12A), we speculate that CTCF might have a significant role in making extensive DNA-DNA contact, looping and regulation of *MOGAT1* transcription and tissue specific expression. Although precise sequential interaction is unclear, we speculate (as illustrated in Fig 12B) that region A binds to regions E and C which brings these regions (E and C) in close proximity for DNA-DNA interaction (Fig 12C).

In the current study we have identified upstream DNA regions which are responsive to PPARα agonist and antagonist adding new regulatory elements for the regulation of *Mogat1*. As mentioned above, *Mogat1* is expressed in tissues not involved in lipid synthesis. *Mogat1* function in these tissues is not characterized and thus this study provides the basis to further explore its function.While developing tissue specific animal models could be labor intensive, a CRISPR/Cas9-based (clustered regularly interspaced short palindromic repeats/CRISPR-associated) genome editing system could be employed to modulate gene expression. This would help identify the function of *MOGAT1* in non-lipid synthesizing tissues. This study also shows that deleting the PPRE or upstream elements (responsive to PPARs) has reduced the *Mogat1* promoter activity by several fold. Recently, Li., et al [41] developed a CRISPR/Cas9 based method for inverting, duplicating, and deleting mammalian DNA fragments of small regulatory elements and large gene clusters which could be very useful to control gene expression. Thus, PPRE sites could be deleted in the Mogat1 promoter using the CRISPR/Cas9 system, thereby inhibiting the binding of PPARs but not affecting the PPARs protein function. This could be an alternative strategy to suppress Mogat1 expression in specific tissues.

## Supporting Information

S1 Fig Amplification of *Mogat1* from normal rat kidney cells (NRK). (TIF)

S2 Fig Mogat1 expression in primary mouse hepatocytes treated with bovine serum albumin-conjugated fatty acids. (TIF)

S3 Fig Amplification of *Mogat1* from rat hepatic tumor cells (HTC). (TIF)

S4 Fig Chromosome conformation capture (3C) assay of rat *MOGAT1* in the rat hepatic tumor (HTC) cells. (TIF)

S1 Table List of rat Mogat1 primers used in this study. (DOCX)

S2 Table List of cis-regulatory elements predicted in the Mogat1 promoter. (DOCX)
