# Supplementary figures and images for: Characterization of the Mouse and Human Monoacylglycerol O-Acyltransferase 1 (Mogat1) Promoter in Human Kidney Proximal Tubule and Rat Liver Cells
Source: PLoS One. 2016 Sep 9;11(9):e0162504. doi: 10.1371/journal.pone.0162504 (PMC5017789; doi:10.1371/journal.pone.0162504)

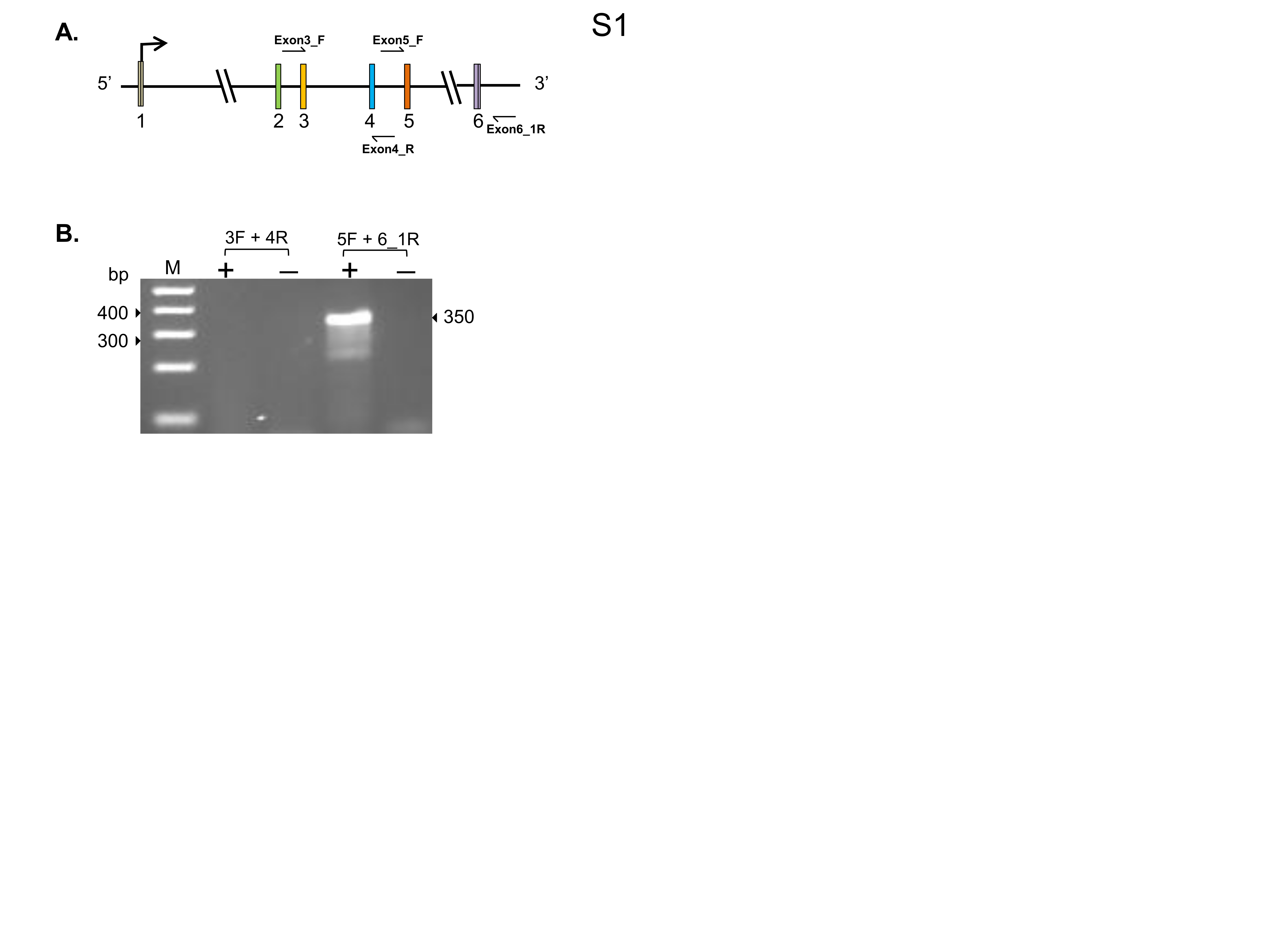

Supplement: S1 Fig — (TIF) [file pone.0162504.s001.tif]

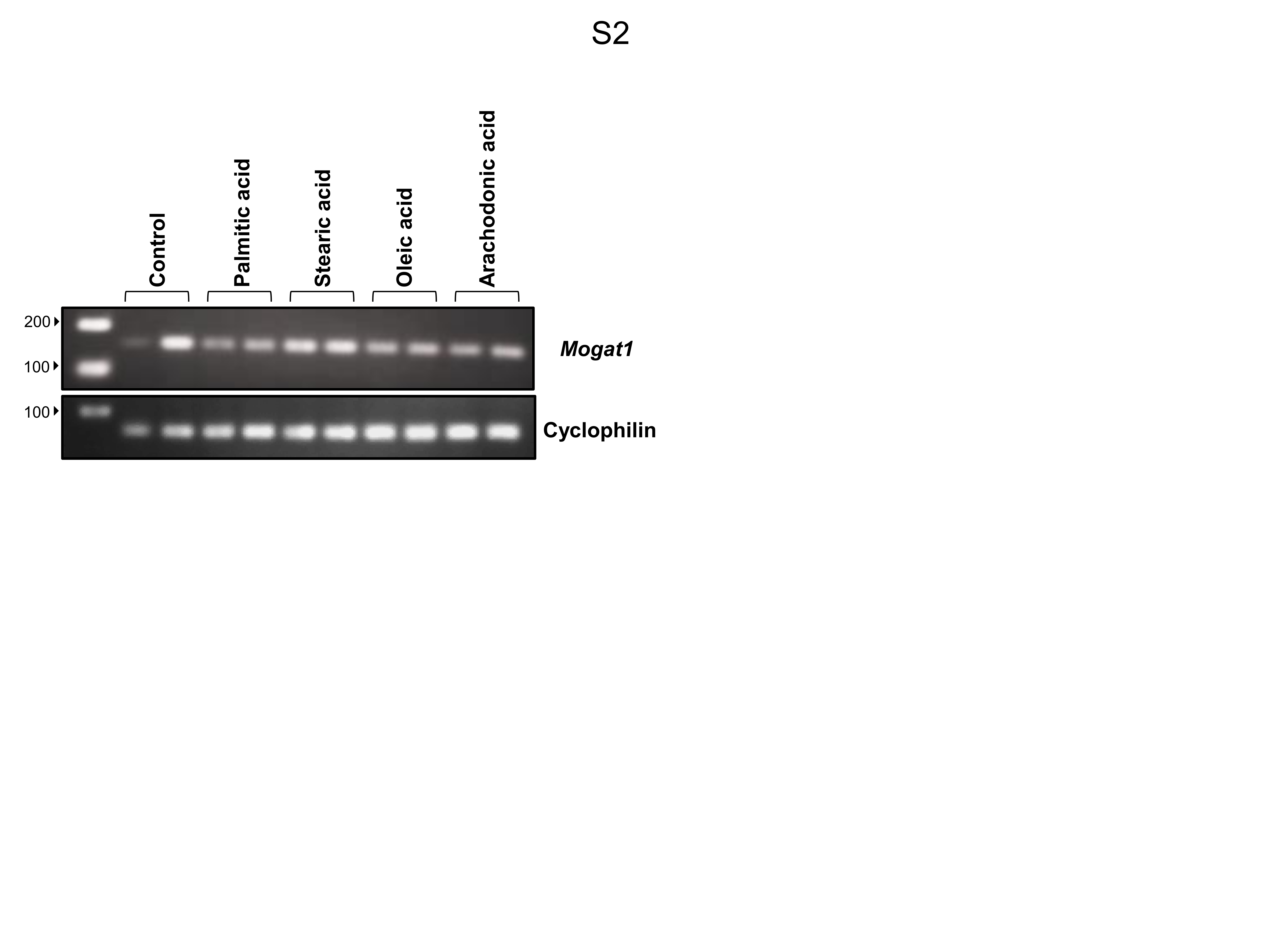

Supplement: S2 Fig — (TIF) [file pone.0162504.s002.tif]

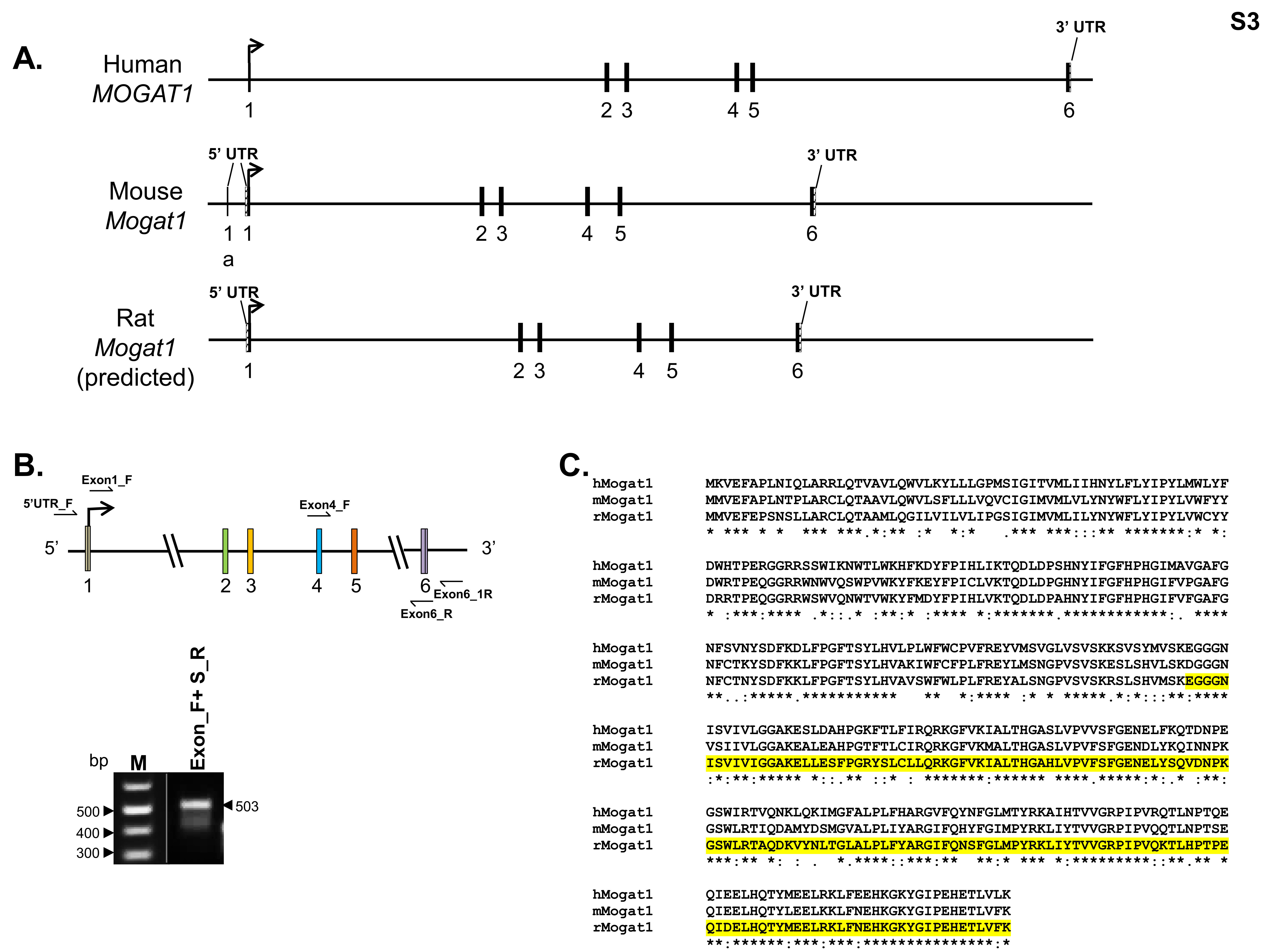

Supplement: S3 Fig — (TIF) [file pone.0162504.s003.tif]

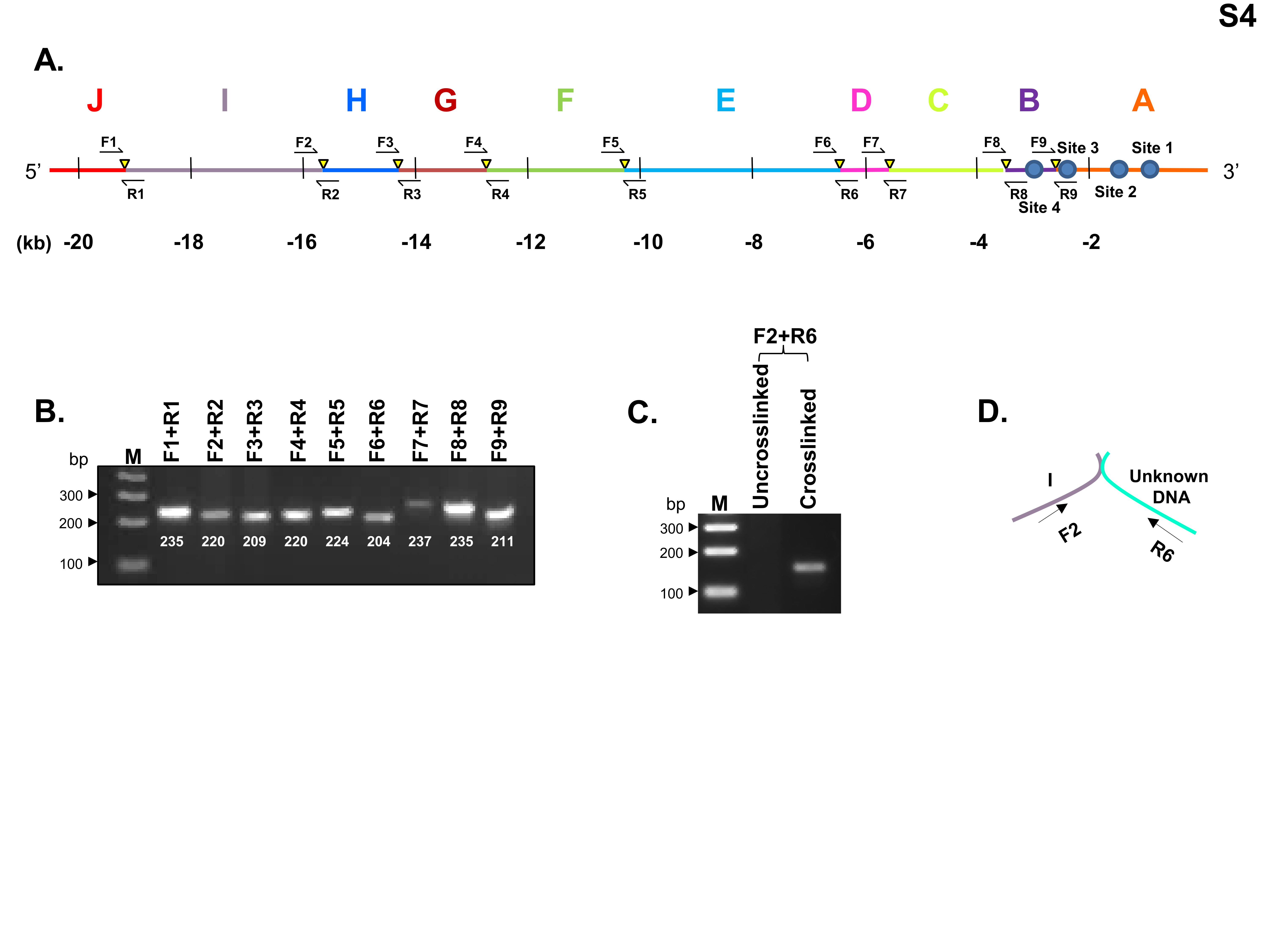

Supplement: S4 Fig — (TIF) [file pone.0162504.s004.tif]
