# Supplementary material for: Characterization of the Mouse and Human Monoacylglycerol O-Acyltransferase 1 (Mogat1) Promoter in Human Kidney Proximal Tubule and Rat Liver Cells
Source: PLoS One. 2016 Sep 9;11(9):e0162504. doi: 10.1371/journal.pone.0162504 (PMC5017789; doi:10.1371/journal.pone.0162504)
Supplement: S1 Table — (DOCX) [file pone.0162504.s005.docx]

**S1 Table. List of rat Mogat1 primers used in this study**

| **Rat Mogat1 primers used in this study** | | |
| --- | --- | --- |
| **3C assay** | | |
|  | **Primer name** | **Primer Sequence** |
| 1 | F1 | AGTACTTTCTCCCACAGCCC |
| 2 | R1 | TGCTGGGATTATAGGCGTGT |
| 3 | F2 | GCCGGTGGTTATTTGGTTGT |
| 4 | R2 | TCCCACCCTTTTCCTTGGAA |
| 5 | F3 | AGGCTGGCATAAACTCCAGA |
| 6 | R3 | TTCGTGTTCCTTCTTCCCCA |
| 7 | F4 | GGACAGAGACCAAATCCTAGTG |
| 8 | R4 | AGTTCAAAACCACAGACTTCAAA |
| 9 | F5 | CCCCAACCACCACTGAATTG |
| 10 | R5 | ACATTCTCTGGCTGTGTCCT |
| 11 | F6 | GACGCAGGTTCTCATCCCT |
| 12 | R6 | TGGTTTGTGGTTGTTGCTGT |
| 13 | F7 | TCCCCTTCGAGTACATCAGT |
| 14 | R7 | AGCTTGGTCCTGGCATAGAG |
| 15 | F8 | GAGCCGAGGACTGAACAGAT |
| 16 | R8 | CCATGGCAGTTGGTTGTGAG |
| 17 | F9 | GGGACTTTGCTGGTTCTTCC |
| 18 | R9 | AACCCCTCAAATGGCAATGG |
