# Supplementary material for: Characterization of the Mouse and Human Monoacylglycerol O-Acyltransferase 1 (Mogat1) Promoter in Human Kidney Proximal Tubule and Rat Liver Cells
Source: PLoS One. 2016 Sep 9;11(9):e0162504. doi: 10.1371/journal.pone.0162504 (PMC5017789; doi:10.1371/journal.pone.0162504)
Supplement: S2 Table — (DOCX) [file pone.0162504.s006.docx]

**S2 Table. List of cis-regulatory elements predicted in the Mogat1 promoter**

| Conserved region | Regulatory element | Canonical sequence | Sequence | Location |
| --- | --- | --- | --- | --- |
| -674 TO -94 | C/EBP | ATTGCGCAAT | GTCTTGTAAT | -661 |
|  | USF-1 | CA(C/T)GTGggggc | CATGTG | -687 |
|  | PPAR | AGGTCANAGGTCA | AGGTCA | -501 |
|  | Sp1 | AGGGGGCGGGGTT | GGGGGCGG | -426 |
|  | YY1 | AANATGGN | GCCCAGGCCC | -225 |
|  | NF-1 | TTGGCTTCCTT | TTGGCTT | -327 |
| -1096 to -1023 | GATA-1 | (A/T)GATA(A/G) | TGATAAA | -1029 |
|  | NF-1 | TTGGCTTCCTT | TTGGCTT | -1045 |
|  | C/EBP | ATTGCGCAAT | GTCTTGTAAT | -1078 |
| -2860 to -2712 | SRF | CC(A/T)6GG | CCTAATTTGG | -2745 |
|  | ATF | GTGACGTACAG, TGACGTCA | AATGACGTAA | -2768 |
|  | C/EBP | ATTGCGCAAT | GTCTTGTAAT | -2802 |
| -5359 to -5188 | GATA-1 | (A/T)GATA(A/G) | TGATAAA | -5233 |
|  | USF | TGTCACGTG | TGACCAGTTT | -5274 |
|  | SRF | CC(A/T)6GG | CCTAATTTGG | -5312 |
